# A prospective analysis of symptom burden for patients with chronic myeloid leukemia in chronic phase treated with frontline second‐ and third‐generation tyrosine kinase inhibitors

**DOI:** 10.1002/cam4.1808

**Published:** 2018-10-14

**Authors:** Alejandro Zulbaran‐Rojas, Huei‐Kan Lin, Qiuling Shi, Loretta A. Williams, Binsah George, Guillermo Garcia‐Manero, Elias Jabbour, Susan O’Brien, Farhad Ravandi, William Wierda, Zeev Estrov, Gautam Borthakur, Tapan Kadia, Charles Cleeland, Jorge E. Cortes, Hagop Kantarjian

**Affiliations:** ^1^ Department of Leukemia The University of Texas MD Anderson Cancer Center Houston Texas; ^2^ Department of Symptom Research The University of Texas MD Anderson Cancer Center Houston Texas

**Keywords:** BCR‐ABL, chronic myeloid leukemia, symptom burden, tyrosine kinase inhibitors

## Abstract

**Background:**

Treatment with tyrosine kinase inhibitors (TKIs) for patients with chronic myeloid leukemia (CML) is effective but needs to continue for several years, possibly indefinitely. Although generally safe, TKI may have hitherto poorly recognized effects in the quality of life (QoL) of such patients.

**Methods:**

We prospectively measured the symptom burden of patients with chronic phase CML enrolled on frontline TKI trials with dasatinib, nilotinib, or ponatinib. A total of 219 patients were enrolled and filled out the MD Anderson Symptom Inventory (MDASI)‐CML questionnaire before the start of therapy and during follow‐up at defined time points of 3, 6, 9, 12, 18, and 24 months.

**Results:**

The median age was 50 years. Longitudinal analysis showed relatively stable symptom severity scores over time. Fatigue was the most common symptom in all three cohorts, both prior to the start of therapy and during therapy, including after achievement of deep molecular remission. Work was the most affected component of daily living. Overall patients tolerated therapy well with improvement of their symptoms from baseline, with few dose reductions related to toxicity or symptomatology. Although 31% of the patients who completed MDASI‐CML achieved complete molecular remission by 24 months of treatment, nearly 90% experienced persistent mild symptoms.

**Conclusion:**

Side effects related to TKIs may impact the quality of life in patients with CML‐CP. Further studies should investigate factors (comorbidities, concomitant medications, dose and schedule, etc) associated with these symptoms and interventions that may improve the patients’ QoL, including treatment discontinuation when safely feasible.

## 1 INTRODUCTION

Treatment with tyrosine kinase inhibitors (TKIs) has improved the long‐term survival of patients with chronic myeloid leukemia (CML) to the point where they now enjoy a life expectancy that is similar to that of the general population.^1^, ^2^, ^3^, ^4^ This however depends on the continued use of TKI for extended periods of time, possibly indefinitely in the majority of patients. This has transformed CML into a manageable chronic disease. Although the average rate of progression with current TKI therapy is approximately 1%‐2% per year,^2^, ^5^ the rate of treatment interruptions, dose adjustments, and discontinuation due to adverse effects are considerably higher.^6^, ^7^ In addition, non‐adherence to therapy is associated with lower rates of early molecular response and, particularly, lack of sustained deeper molecular responses. This may in turn result in lower probability of overall and progression‐free survival, and of successful treatment‐free remission.^8^, ^9^ Thus, exploring the long‐term consequences of exposure to TKI has become increasingly important. These include the possible development of secondary comorbidities including renal^10^ or cardiovascular complications.^11^ In addition, the chronic exposure to TKI may have effects on the quality of life (QoL) of patients and impact their activities of daily living in ways not hitherto well described.

The purpose of this study was to investigate the symptom burden associated with frontline TKI therapy and its impact on QoL and treatment outcomes.

## 2 PATIENTS AND METHODS

### 2.1 Patient enrollment and study design

A total of 219 patients with newly diagnosed CML‐CP were enrolled in prospective clinical trials phase II of frontline therapy with second‐generation (dasatinib [NCT00254423] and nilotinib [NCT00129740]) or third‐generation (ponatinib [NCT01570868]) TKIs agreed to participate in this optional component of their therapeutic studies from April 2005 to May 2015. Patients consented and were enrolled in frontline therapy studies with dasatinib (n = 104; starting dose 100 mg daily), nilotinib (n = 82; starting dose 400 mg twice daily), and ponatinib (n = 33; starting dose 45 mg/d in 27 and 30 mg/d in 6). The study design and general results of these studies have been previously reported.^1^, ^12^, ^13^ QoL was evaluated using the MD Anderson Symptom Inventory (MDASI‐CML) questionnaire, which was completed by the patients at the time of enrollment on the corresponding clinical trial and prior to start of therapy.^14^ The MDASI‐CML questionnaire was subsequently completed during follow‐up visits at 3, 6, 9, 12, 18, and 24 months from the start of therapy.

The eligibility was similar for all trials and generally included diagnosis of Ph‐positive or Bcr‐Abl‐positive CML in early chronic phase CML (ie, time from diagnosis <12 months); except for hydroxyurea, patients must have received no or minimal prior therapy (defined as <1 month of prior IFN‐a and/or FDA‐approved TKI); age ≥16 years (age >18 years to participate in optional symptom burden assessment); ECOG (Eastern Oncology Cooperative Group scale) performance status of 0‐2; adequate organ function (defined as total bilirubin <1.5× ULN, SGPT <2.5× ULN, creatinine <1.5× ULN); and, to participate in optional symptom burden assessment, patients should be able to speak and read English. Patients meeting any of the following criteria were excluded: NYHA cardiac class 3‐4 heart disease; cardiac symptoms (uncontrolled angina, suspected congenital long QT syndrome, history of ventricular arrhythmias, prolonged QTc interval, uncontrolled hypertension, congenital bleeding disorders, patients under risk to develop torsades des pointes due to drug treatment, LVEF <45%, cardiac pacemaker, ST depression of >1 mm, myocardial infarction within 1 year prior to starting drug treatment); uncontrolled psychiatric disorders (psychosis, major depression, bipolar disorders); pregnant or breastfeeding women; patients in late chronic phase (ie, time for diagnosis >12 months), accelerated or blast phase; patients with severe or uncontrolled medical disease (ie, diabetes, chronic renal disease, active uncontrolled infection); patients with known chronic liver disease (ie, chronic active hepatitis, cirrhosis), or patients with known diagnosis of HIV infection.

The studies were conducted in accordance with the Declaration of Helsinki. All patients enrolled signed an informed consent document approved by the Institutional Review Board.

### 2.2 Response to treatment

Patients were evaluated for response to therapy every 3 months for the first year and every 6 months thereafter. BCR‐ABL transcripts were measured using real‐time reverse transcription‐polymerase chain reaction (PCR) analysis on peripheral blood and/or bone marrow aspirate. A complete cytogenetic response (CCyR) is defined as PH+ 0% (grossly equivalent to BCR/ABL1/ABL1 transcripts <1% in the international scale [IS]). A major molecular response (MMR) is defined as BCR‐ABL1/ABL1 transcripts ≤0.1% IS,^8^, ^15^ MR4 as BCR‐ABL1/ABL1 ≤0.01% IS, and MR4.5 as BCR‐ABL1/ABL1 ≤0.0032% IS.^16^ Undetectable transcript (herein reported as “CMR”) was reported when no transcripts were detected with at least 100 000 copies of ABL control gene.^4^ An optimal response is defined as PH+ <35% and/or BCR/ABL1 ≤10% at 3 months, PH+ <0% (CCyR) and/or BCR/ABL1 ≤1% at months 6, and BCR/ABL1 ≤0.1% (MMR) at 12 months or later.^17^

### 2.3 Symptom assessment

Patients were asked to fill the MDASI‐CML questionnaire. The MDASI‐CML module consists of a questionnaire filled by the patients to assess the severity of 20 symptoms. These include 13 cancer‐related symptoms (fatigue, difficulty remembering/memory, nausea, disturbed sleep, vomiting, distress, pain, dyspnea, appetite, drowsiness, dry mouth, numbness, and sadness) and 7 CML‐specific symptoms (muscle soreness/myalgia, swelling of extremities, bruising/bleed, skin rash, malaise, headache, and diarrhea). The questionnaire also includes six questions related to interference with various aspects of life (WAW: Work, general Activity, Walking, and REM: Relations with others, Enjoyment of life, Mood).^14^ All items were scored by the patient from 0 (not present) to 10 (worst) at each time point, and classified as mild (scores 1‐4), moderate (scores 5‐6), or severe (scores 7‐10). This questionnaire was psychometrically validated as a reliable, valid and sensitive tool to collect CML patients’ perspectives on their symptoms related to treatments.^14^

### 2.4 Statistical analysis

Descriptive analysis was used to present patient characteristics. Mean and standard deviation were used to describe the symptom score at each time point. To estimate the change in symptom severity over time, we used mixed‐effects models with a random subject effect to approximate the change in each symptom item.^18^ Those who started their MDASI‐CML assessments at the beginning of TKI treatment were included in models, for an unbiased understanding of symptom burden change over time. Symptom severity scores were treated as continuous variables, and the quadratic time variables (month) were included. The interpretation focuses on the average change in the outcome along the 0‐10 scale. Observations within 24 months were included in this analysis. We used SAS 9.3 (SAS Institute Inc, Cary, NC) for analysis, and the significant levels were set as 0.05.

## 3 RESULTS

All eligible patients were approached. Two of the protocols (Dasatinib and Nilotinib) began enrolling patients before the MDASI‐CML had been developed. After MDASI‐CML was developed, it was added to these two protocols. The MDASI‐CML was part of Ponatinib protocol from the onset of the trial. A total of 219 patients were included and followed over a median of 54 months (range, 8‐112) from the start of therapy. These represent 72% of all patients treated in these studies. Their median age was 50 years (range, 16‐86) and 57% were male. Demographic and baseline patient characteristics are summarized in Table 1. At baseline, fatigue, dry mouth, and drowsiness were the symptoms with the highest mean scores, while work was the most affected daily activity at baseline.

**Table 1:** Overall and each cohort baseline demographic and clinical characteristics

Demographic or characteristic	Median [range] or no. of patients (%)			
	Overall N = 219	Dasatinib N = 104	Nilotinib N = 82	Ponatinib N = 33
Age, y	50 [16‐86]	47 [16‐82]	50 [23‐86]	50 [28‐74]
Sex, male	126 (57)	63 (60)	48 (46)	15 (45)
Race, white	175 (80)	88 (84)	66 (63)	21 (63)
Follow‐up, mo	54 [8‐112]	54 [9‐110]	68 [8‐112]	22 [9‐32]
BMI	28 [18‐57]	30 [18‐57]	29 [19‐54]	28 [18‐46]
Sokal risk^a^				
Low	154 (70)	76 (73)	55 (67)	23 (69)
Intermediate	46 (21)	21 (20)	20 (24)	5 (15)
High	17 (7)	7 (0.6)	5 (0.6)	5 (15)
WBC (×10^9^/L)	46 [1‐342]	46 [1‐295]	51 [2‐342]	40 [3.8‐193.7]
PB blast, %	0 [0‐7]	0 [0‐4]	0 [0‐7]	0 [0‐3]
BM blast, %	1.6 [0‐10]	2 [0‐8]	1.6 [0‐4]	1 [0‐10]
PB basophils, %	3.8 [0‐19]	3.5 [0‐17]	3.8 [0‐19]	3.8 [0‐16]
BM basophils, %	2 [0‐13]	2 [0‐11]	2.5 [0‐13]	2 [0‐8]
Splenomegaly, cm	0 [0‐30]	0 [0‐23]	0 [0‐30]	0 [0‐13]
CE at diagnosis	0 [0‐2]	0	0	0 [0‐2]
Dose, mg		100 daily	800 daily	45 daily

BM, bone marrow; PB, peripheral blood, WBC, white blood cells.

^a^Predicts survival for CML based on age, spleen size, platelet count, and % myeloblasts. Low corresponds to Sokal score of 0.8; intermediate corresponds to Sokal score of 0.8 to 1.2; and high corresponds to Sokal score of 1.2.

### 3.1 Response to therapy

Overall response to therapy over the first 24 months corresponding to the timeline of symptom assessment is depicted in Table S1. By 3 months of therapy, more than 80% of patients had achieved transcript levels <10%, which are associated with the best long‐term outcome. An MMR was achieved by 75% of patients at 9 months of therapy, MR4 by 50% of patients at 12 months, MR4.5 by 25% of patients at 18 months, and a CMR by 31% of patients at 24 months. In all three treatment groups (dasatinib, nilotinib, and ponatinib), there was a steady improvement of response over time. In addition, more than 90% of patients maintained a CCyR during the MDASI‐CML assessment period. There were no significant differences in the response rates between the cohorts with different TKI, and only 5% of all patients did not have any cytogenetic response by 24 months.

### 3.2 Longitudinal development of symptom score by all cohorts over 24 months

**Figure 1:**
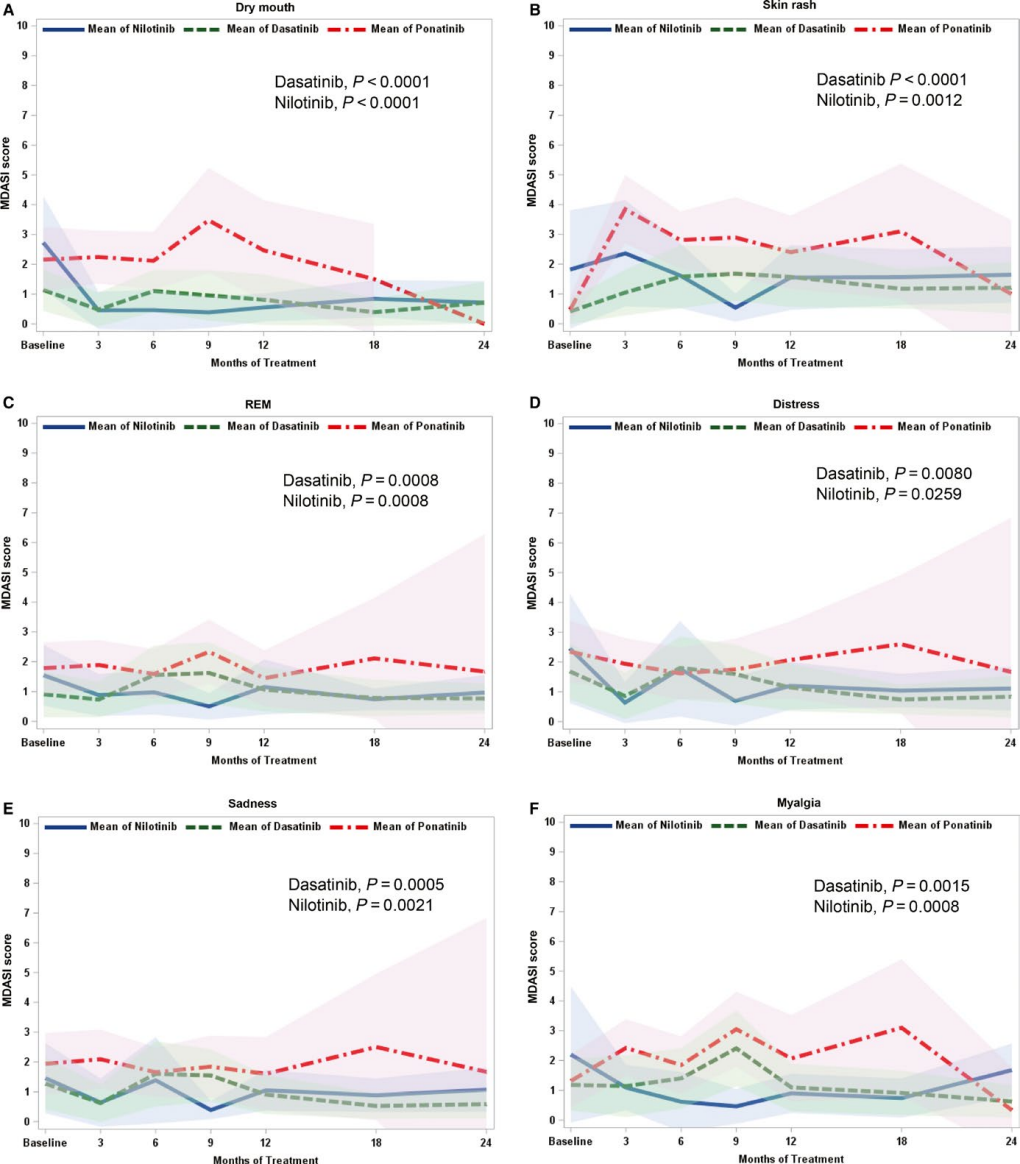
Specific symptom burden for ponatinib, dasatinib, and nilotinib cohorts over 24 mo of therapy. Longitudinal analysis. MDASI, MD Anderson Symptom Inventory; Comparison between all cohorts. Panels A, dry mouth (dasatinib and nilotinib, *P* < 0.0001), B, skin rash (dasatinib *P* < 0.0001, nilotinib *P* = 0.0012), C, relations, enjoyment, and mood (dasatinib and nilotinib, *P* = 0.0008), D, distress (dasatinib *P* = 0.0080, nilotinib *P* = 0.0259), E, sadness (dasatinib *P* = 0.0005, nilotinib *P* = 0.0021), and F, myalgia (dasatinib *P* = 0.0015, nilotinib *P* = 0.0008), over 24 mo of therapy

Patients were assessed with MDASI at the specified intervals. Overall, fatigue had the highest mean scores (2.63, standard deviation [SD] = 2.57) throughout the observation period for all three cohorts. Regarding interference with daily activities, WAW was the most affected variable (mean = 1.5, SD = 2.13). When analyzing the scores averaged throughout the study period, the top overall five symptoms for all patients combined were fatigue, drowsiness, disturbed sleep, skin rash, and difficulty remembering. There was no significant difference for fatigue mean scores between the three cohorts (*P* > 0.05), but ponatinib had the highest numerical scores in the majority of symptoms by 24 months (Table 2). When comparing mean scores between cohorts, patients on ponatinib had higher significant scores of disturbed sleep (*P* = 0.02) than patients on dasatinib, and of malaise (*P* = 0.0113), swelling of extremities (*P* = 0.0003), pain (*P* = 0.0086), shortness of breath (*P* = 0.010), and WAW (*P* = 0.0285) than patients on nilotinib. In addition, skin rash (*P* < 0.0001 vs dasatinib,* P* = 0.0012 vs nilotinib), muscle cramps (*P* = 0.0015 vs dasatinib, *P* = 0.0008 vs nilotinib), dry mouth (*P* < 0.0001), distress (*P* = 0.008 vs dasatinib, *P* = 0.028 vs nilotinib), and REM (*P* = 0.0008) scores were significantly higher with ponatinib compared to both dasatinib and nilotinib cohorts (Figure 1). Patients on nilotinib had significant higher scores of disturbed sleep (*P* = 0.026), diarrhea (*P* = 0.009), and swelling of extremities (*P* = 0.02) than patients on dasatinib, while pain had a higher score among patients on dasatinib (*P* = 0.03) compared to those on nilotinib. Importantly, the majority of patients who completed MDASI‐CML experienced persistent mild symptoms at each time point.

**Table 2:** Top 5 symptoms over 24 mo from baseline by treatment cohort

Overall N = 219		Dasatinib N = 104		Nilotinib N = 82		Ponatinib N = 33	
Item	Mean (SD)	Item	Mean (SD)	Item	Mean (SD)	Item	Mean (SD)
Fatigue	2.63 (2.57)	Fatigue	2.39 (2.47)	Fatigue	2.47 (2.24)	Fatigue	3.03 (2.89)
Drowsiness	2.08 (2.31)	Drowsiness	1.80 (2.21)	Disturbed sleep	2.18 (2.29)	Skin rash	2.45 (2.73)
Disturbed sleep	2.02 (2.47)	Disturbed sleep	1.58 (2.30)	Drowsiness	2.14 (2.17)	Disturbed sleep	2.37 (2.72)
Skin rash	1.74 (2.35)	Pain	1.52 (2.36)	Difficulty remembering	1.78 (1.97)	Drowsiness	2.34 (2.52)
Difficulty remembering	1.65 (2.03)	Difficulty remembering	1.65 (1.95)	Skin rash	1.57 (2.24)	Dry mouth	2.29 (2.85)
Inference with life							
WAW	1.5 (2.13)	WAW	1.37 (2.01)	WAW	1.16 (1.82)	WAW	1.94 (2.44)
REM	1.28 (1.84)	REM	1.05 (1.7)	REM	0.95 (1.36)	REM	1.83 (2.21)

Longitudinal analysis: REM, relations with others, enjoyment of life, and mood; SD, standard deviation; WAW, working with others, general activity, and walking.

Overall top 5 symptoms for all patients combined are represented in column 1. The next three columns represent the top 5 symptoms for each cohort, separately.

### 3.3 Symptom burden assessment with individual TKI over specific time points

#### 3.3.1 Dasatinib

The overall longitudinal mean scores of the top five symptoms while on dasatinib therapy over 24 months from baseline are depicted in Table 2. The five symptoms with the highest mean severity had mostly low scores (ie, scores 0‐3). Although fatigue had the highest mean score (2.39, SD = 2.47), only 6 (6%) patients had a dose reduction by 24 months for this adverse event (2 of them concomitantly with headaches and one with depression) (Table 3).

**Table 3:** Non‐hematologic adverse events and dose reductions by treatment cohort by time point

	Months						Total n (%)
	3 n (%)	6 n (%)	9 n (%)	12 n (%)	18 n (%)	24 n (%)	
Dasatinib	N = 8/103	N = 5/101	N = 2/100	N = 6/100	N = 5/100	N = 3/99	N = 29/104
Pleural effusion	5 (4.85)	4 (3.96)	1 (1)	4 (4)	2 (2)	3 (3.03)	19 (18.26)
Shortness of breath	1 (0.97)	1 (0.99)	0	1 (1)	4 (4)	2 (6.06)	9 (8.65)
Fatigue	0	1 (0.99)	0	2 (2)	0	3 (3.03)	6 (5.76)
Edema	3 (2.91)	0	0	1 (1)	1 (1)	1 (1.01)	6 (5.76)
Head ache	2 (1.94)	2 (1.98)	1 (1)	0	0	1 (1.01)	6 (5.76)
	11 (10.6)	8 (7.9)	2 (2)	8 (8)	7 (7)	10 (10.1)	46 (44.23)
Nilotinib	N = 8/82	N = 5/82	N = 2/81	N = 1/81	N = 2/80	N = 2/78	N = 20/82
Hyperbilirubinemia	3 (3.65)	2 (2.4)	1 (1.23)	0	0	0	6 (7.31)
Fatigue	1 (1.21)	2 (2.4)	1 (1.23)	0	0	1 (1.28)	5 (6.09)
Hepatotoxicity (ALT/AST)	1 (1.21)	3 (3.65)	0	0	0	1 (1.28)	5 (6.09)
Skin rash	2 (2.4)	1 (1.21)	1 (1.23)	1 (1.23)	0	0	5 (6.09)
Nausea/vomiting	1 (1.21)	1 (1.21)	0	0	2 (2.5)	0	4 (4.87)
	8 (9.7)	9 (10.9)	3 (3.7)	1 (1.2)	2 (2.5)	2 (2.56)	25 (30.4)
Ponatinib	N = 7/33	N = 9/30	N = 3/27	N = 3/23	N = 11/20	N = 0/16[Link]	N = 33/33
Increased cardiovascular risk	0	4 (13.3)	4 (14.81)	3 (13.04)	7 (35)	0	18 (54.54)
Pancreatitis	7 (21.21)	2 (6.66)	1 (3.7)	0	1 (5)	0	11 (33.33)
Hypertension	0	0	0	2 (8.69)	2 (10)	0	4 (12.12)
Skin rash	0	3 (10)	0	1 (4.34)	0	0	4 (12.12)
Fatigue	0	2 (6.66)	0	0	0	0	2 (6.06)
	7 (21.21)	11 (36.6)	5 (18.51)	6 (26)	10 (50)	0	39 (118.8)
Total events per month	26 (12)	28 (13.1)	10 (4.8)	15 (7.3)	19 (9.5)	12 (6.2)	110 (50.2)
Total patients per month	N = 23/218	N = 19/213	N = 7/208	N = 10/204	N = 18/200	N = 5/193	N = 82/219

N, total number of patients with dose reduction; n, number of events.

Ponatinib cohort had study termination after 18 mo of treatment. By 24 mo, all patients were on another TKI therapy.

**Figure 2:**
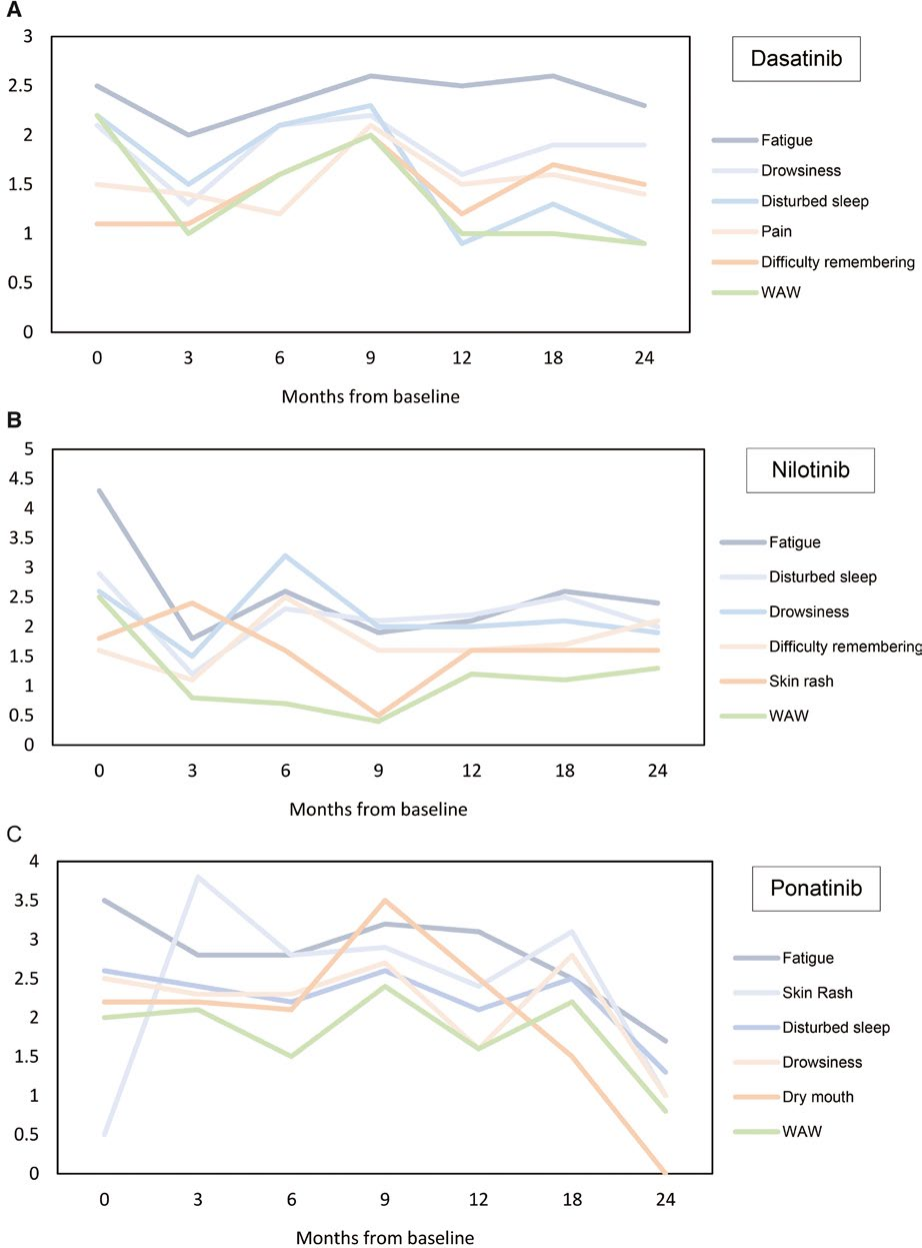
Evolution of top five symptoms and interference with life mean scores for each treatment cohort over 24 mo. Cross‐sectional analysis. Top five mean score symptoms and interference with life. Panels A, dasatinib cohort, B, nilotinib cohort, and C, ponatinib cohort, over time of assessment

In a cross‐sectional analysis, 92% of patients in this cohort that had achieved an optimal response by 3 months of therapy reported a significant drop below baseline values in their top five symptom mean scores. Thereafter, there were two peak mean scores reported, at 9 and 18 months of therapy, respectively (Figure 2A). During the first peak, muscle cramps reached to the top five symptoms with a significant higher score than patients on nilotinib (*P* = 0.028). In addition, the mean scores for drowsiness, fatigue, and swelling of extremities were higher at 24 months (*P* > 0.05) compared to the significant drop previously observed at 3 months of therapy. However, the majority of symptoms stabilized or decreased slightly to levels slightly below baseline values across all variables.

**Figure 3:**
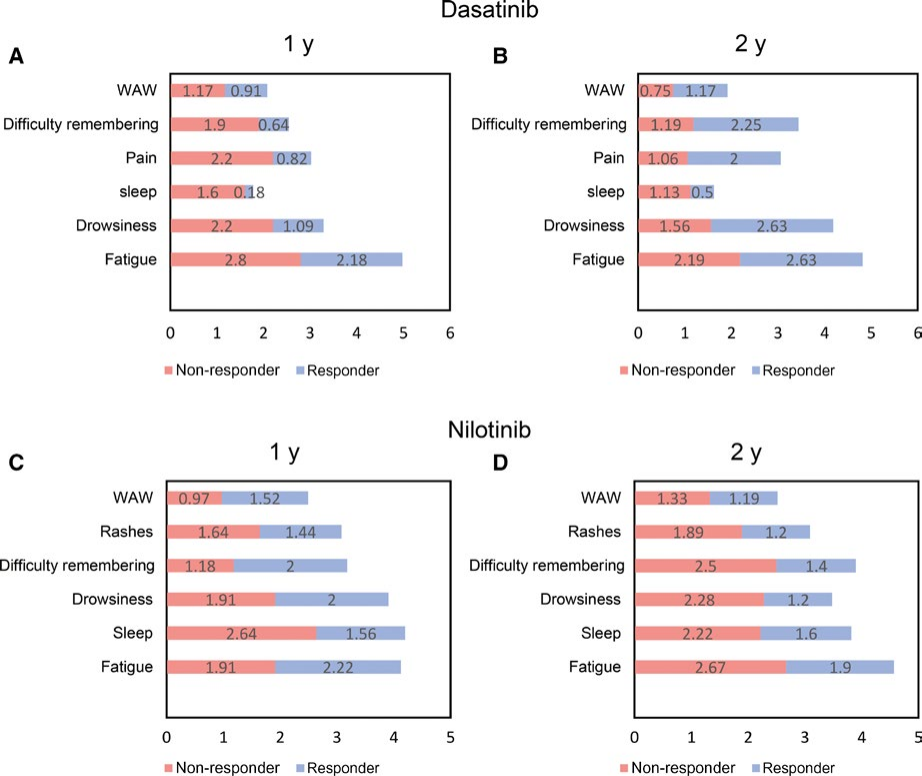
Top five symptom mean score comparison between responders and non‐responders with dasatinib and nilotinib. Stacked bar graphs representing symptom mean score comparison between responder and non‐responder groups (≥MMR at 1 y; ≥CMR at 2 y). Panel A, Dasatinib at 1 y from baseline; Panel B, Dasatinib at 2 y from baseline; Panel C, nilotinib at 1 y from baseline; Panel D, nilotinib at 2 y from baseline. WAW, work, activity, and walk; REM, relations with others, enjoyment of life, and mood; Sleep, disturbed sleep; Rashes, skin rash. The majority of symptoms did not show statistical significance

When comparing symptom scores by level of response (responders vs non‐responders), patients reaching target therapeutic goals (ie, optimal response) had better mean symptom scores over the first year of treatment than those without such responses. Except for diarrhea, none of the individual symptoms were significantly higher in the non‐responder group. Interestingly, mean scores for responders were higher at 24 months of treatment (Figure 3A,B). At 1 year, the median dose of dasatinib was 100 mg/d for responders (ie, MMR at 12 months; mean = 89) and non‐responders (mean = 91.7) alike. However, at 2 years, the median dose for responders (ie, MR4.5) was 80 mg/d (mean = 78.8), while for non‐responders stayed in 100 mg/d (mean = 87.05). Although there was a trend for some symptoms having a worse score among responders compared to non‐responders, the difference was not statistically significant and likely the result of small numbers in this subset analysis.

#### 3.3.2 Nilotinib

As with the dasatinib cohort, fatigue (mean = 2.47, SD = 2.24) and WAW daily interference (mean = 1.16, SD = 1.82) were the most prevalent symptoms in longitudinal analysis (Table 2) with a high number of patients experiencing fatigue at a mild‐to‐moderate level (ie, scores 3‐6). Although fatigue had numerically higher mean scores than the dasatinib cohort, a similar low number of patients (6%) had experienced dose reduction by 24 months due to fatigue (alone or in conjunction with other adverse events such as headache, nausea, and dry mouth) (Table 3).

In a cross‐sectional analysis, all top five symptom mean scores peaked at 6 months from the start of therapy (ie, 3 months earlier than in the dasatinib cohort) (Figure 2B), a time when 90% of patients had already achieved an optimal response. After 1 year of therapy, disturbed sleep was the worst symptom with significant higher mean scores than the dasatinib cohort (*P* = 0.042). Similar to what was seen with dasatinib, patients that had reached optimal response at 18 months had higher mean symptom scores in four of the top five symptoms. However, these changes in symptom scores did not reach statistical significance (*P* > 0.05), and mean scores stabilized or decreased slightly to levels below baseline across all variables over 24 months of therapy.

The trends in mean scores were in the opposite direction seen in the dasatinib cohort. By 1 year of treatment, 45% of overall symptom scores were worse in patients reaching target therapeutic goals in comparison with the group who did not attain such response, but better mean symptom scores were seen in responders at 24 months of treatment (Figure 3C,D). At 1 year from start of therapy, the median dose for both groups was similar (responders, median 800 mg/d [mean = 717.5]; non‐responders, median 800 mg/d [mean = 629.2]) and remained stable at 2 years (responders median 800 mg/d [mean = 700]; non‐responders median 800 mg/d [mean = 637]). Distress and sadness were significantly higher (*P* < 0.05) at 9 months of treatment for the non‐responders; there was no difference in other individual symptom scores.

#### 3.3.3 Ponatinib

In longitudinal analysis, fatigue (mean = 3.03, SD = 2.89) and WAW daily interference (mean = 1.94, SD = 2.44) were the highest scoring symptoms (Table 2) with a high number of patients experiencing both at a mild‐to‐moderate level (ie, scores 3‐6). In cross‐sectional analysis, the majority of mean symptom scores in the ponatinib cohort peaked earlier (at 3 months) or stayed at the same baseline value with a subsequent trend for improvement over time. For example, skin rash and dry mouth, which had significantly higher scores at 3 months of therapy compared to dasatinib and nilotinib cohorts (*P* < 0.0001), improved over time and did not make it to the top five symptoms by 24 months. Similarly, among interference with daily living, WAW had a high mean score in the initial months of treatment but decreased significantly by 24 months. Moreover, as was seen in the dasatinib cohort, patients on ponatinib had a peak around 9 months of therapy (second peak for ponatinib cohort). Several mean score symptoms (muscle cramps *P* = 0.005, malaise *P* = 0.032, shortness of breath *P* = 0.011, dry mouth *P* = 0.003, pain *P* = 0.046, sadness *P* = 0.031, swelling *P* = 0.006, REM *P* = 0.019, and WAW *P* = 0.021) were significantly higher compared to the nilotinib cohort. Importantly in this cohort, because of concerns of possible cardiotoxicity, all patients had an early elective dose reduction (15 mg/daily, respectively) at approximately 18 months of therapy, and the trial was terminated prematurely at the recommendation of regulatory authorities. Because of this, the number of patient evaluable at later time points was small. By 18 months of therapy, the ponatinib cohort had the highest overall interference with daily living scores compared to the other therapy groups (*P* = 0.23 vs nilotinib, *P* = 0.028 vs dasatinib). Other symptom scores were also significantly higher than the dasatinib and nilotinib cohorts (eg, muscle cramps, sadness, and distress [*P* < 0.05]). Still, the ponatinib cohort experienced the most significant decrease of the top 5 mean scores to scores below baseline at 24 months (Figure 2C).

Correlations between symptom score and response cannot be analyzed as nearly all patients had reached optimal response by 3 months of therapy.

### 3.4 Top symptom scores for responders and non‐responders

Fatigue, disturbed sleep, and drowsiness were the most severe symptoms that coincide in the longitudinal analysis for both the overall population and when analyzing each cohort separately. However, overall patients achieving deep molecular response (ie, MR4) after 1 year had better symptomatology score in comparison with patients not achieving it (Table 4). This held true also for interference with activities of daily life. Even though non‐responders had a worse mean symptom score, there was no significant difference in the majority of symptoms (*P* > 0.05). Furthermore, mean symptom scores showed a downward trend over the course of therapy whether patients achieved a CMR or not.

**Table 4:** Overall top 5 symptom mean score comparison between responders and non‐responders by time

PCR IS	9 mo			12 mo			18 mo			24 mo		
	Non‐Responder N = 8 mean	Responder N = 28 Mean	*P*	Non‐Responder N = 21 mean	Responder N = 20 mean	*P*	Non‐Responder N = 33 mean	Responder *P* N = 15 mean		Non‐Responder N = 35 mean	Responder N = 18 mean	*P*
Symptom												
Fatigue	3.00	2.14	0.366	2.33	2.20	0.831	2.85	2.00	0.270	2.44	2.22	0.780
Drowsiness	2.88	1.93	0.331	2.05	1.50	0.331	2.27	1.47	0.242	1.94	1.83	0.866
Sleep	3.75	1.82	0.056	2.14	0.80	0.022	2.18	1.33	0.231	1.71	1.11	0.329
Skin rash	1,75	1.11	0.378	1.95	1.15	0.258	1.41	1.29	0.842	1.44	1.44	0.996
Difficulty remembering	2.13	1.78	0.677	1.52	1.25	0.687	1.94	1.20	0.192	1.88	1.78	0.871
Interference with life												
WAW	2.00	1.23	0.441	1.06	1.18	0.834	1.10	0.90	0.660	1.06	1.18	0.828
REM	2.10	1.00	0.195	1.27	0.93	0.553	0.78	0.71	0.858	0.75	1.12	0.355

REM, relations with others, enjoyment of life, and mood; WAW, working with others, general activity, and walking.

At 9 mo, non‐responders were defined as >0.1 PCR IS % transcripts, while responders were defined as ≤0.1 PCR IS % transcripts; at 12 mo, non‐responders were defined as >0.01 PCR IS % transcripts, while responders were defined as ≤0.01 PCR IS % transcripts; at 18 mo, non‐responders were defined as >0.0032 PCR IS % transcripts, while responders were defined as ≤0.0032 PCR IS % transcripts; at 24 mo, non‐responders were defined as >0.00001 PCR IS % transcripts, while responders were defined as ≤0.00001 PCR IS % transcripts.

### 3.5 Toxicity

Patients were screened for adverse events at the same intervals when they filled the MDASI‐CML survey. Non‐hematologic adverse events are depicted in Table 3. Overall, 37% patients experienced at least one dose adjustment during the time of assessment. The highest number of dose adjustments for all cohorts was at 3 months of therapy (N = 23). Fatigue was the most common adverse event that led to dose reduction in all cohorts (by itself or concomitantly with other adverse events; n = 13). Nearly one fifth of patients on dasatinib had at least one dose reduction, most commonly due to pleural effusion, and 3 of these patients had to discontinue therapy by 24 months. In the nilotinib cohort, dose adjustments occurred mostly before 1 year of therapy. Hyperbilirubinemia was one of the most common non‐hematologic adverse event, with 6% of dose reductions, and three patients had to discontinue therapy due to liver toxicity. In the ponatinib cohort, pancreatitis was the most common cause of dose reduction early during the course of therapy (N = 10), while increased risk of cardiovascular events led to a preemptive dose reduction in all patients at a later time. By 24 months, the entire cohort had to come off therapy with ponatinib due to study termination with a 62% of patients achieving CMR.

### 3.6 Dose reduction and symptom scores

**Figure 4:**
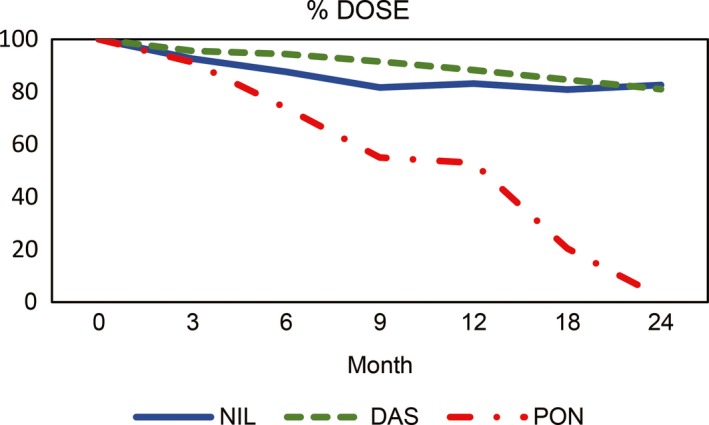
Percentage of dose intensity for each treatment cohort by specific time points where the starting point is the full starting dose and subsequent time points reflect the mean percent dose of the starting dose. Das: dasatinib; Pon: ponatinib; Nil: nilotinib. Ponatinib cohort has terminated this study between 18 and 20 mo of therapy due to severe cardiovascular complications. Eventually, dose intensity went down to 0% at 24 mo

For the total population, there was a reduction in the starting dose from baseline to a mean percentage of 77.2% (SD = 34.16) by 24 months of therapy. Patients on dasatinib and nilotinib had similar dose reductions (dasatinib mean = 81.03%, SD = 30.75; nilotinib mean = 82.6%, SD = 28.07; respectively) at the end of the observation period; patients on ponatinib had a higher dose reduction by 18 months (mean = 20%, SD = 16.46), in part due to the mandated preemptive dose reduction to minimize cardiovascular events (Figure 4).

We analyzed the correlation of dose reduction with MDASI mean scores at different time points (Table 5). There was a significant negative correlation of dose reduction and symptom burden in the total patient population for nausea (*P* = 0.013, *r* = −0.086), dry mouth (*P* = 0.001, *r* = −0.15), vomiting (*P* = 0.007, *r* = −0.01), skin rash (*P* = 0.05, *r* = −0.06), and muscle cramps (*P* = 0.002, *r* = −0.12) by 24 months. However, the overall scores reported a small effect size (*r* < 0.3) (Table S2).

**Table 5:** Correlation between mean percentage dose and top 5 symptom mean scores at specific time points

	Dasatinib						Nilotinib						Ponatinib					
	6		12		18		6		12		18		6		12		18	
	obs = 48 [dose = 94%]		obs = 56 [dose = 88%]		obs = 74 [dose = 85%]		obs = 12 [dose = 88%]		obs = 21 [dose = 83%]		obs = 46 [dose = 81%]		obs = 50 [dose = 74%]		obs = 19 [dose = 53%]		obs = 21 [dose = 20%]	
	*r*	*P*	*r*	*P*	*r*	*P*	*r*	*P*	*r*	*P*	*r*	*P*	*r*	*P*	*r*	*P*	*r*	*P*
Fatigue	−0.07	0.63	0.35	0.009	0.36	0.002	−0.70	0.011	−0.006	0.98	−0.1	0.49	0.15	0.29	0.21	0.35	0.6	0.006
Drowsiness	0.05	0.72	0.28	0.04	0.32	0.005	−0.95	<.0001	−0.21	0.35	−0.23	0.11	0.24	0.09	0.29	0.22	0.62	<.0001
Sleep disturbance	−0.03	0.84	0.25	0.068	0.28	0.015	−0.34	0.29	−0.09	0.68	−0.24	0.1	0.15	0.28	−0.003	0.99	0.38	0.19
Pain	−0.06	0.67	0.17	0.19	0.20	0.08	0.09	0.77	−0.26	0.24	−0.19	0.18	−0.002	0.99	0.13	0.54	0.37	0.12
Difficulty remembering	0.07	0.63	0.19	0.15	0.29	0.01	−0.78	0.002	−0.36	0.1	−0.4	0.007	0.35	0.01	0.1	0.66	0.5	0.36

Obs, observations.

Six, 12, and 18 mo were the most relevant time points with large effect size and significant correlation. The resting time points (3, 9, and 24 mo) did not show any significant correlation between mean dose percentage and symptom scores.

## 4 DISCUSSION

Since the advent of TKI therapy in 2000, the mortality from CML has decreased from 10%‐20% to 1%‐2%, while it is projected that in 2018, nearly 8,430 patients will be diagnosed with CML in the United States.^19^ The estimated prevalence of CML in 2015 was estimated at 70 000 in 2010, and predicted to reach 181 000 in 2050 as a result of the improved overall survival of patients treated with TKI.^20^, ^21^ Recent analysis showed that 5‐year survival is comparable to general population if a complete cytogenetic response or deeper (ie, molecular) is attained within 1 year of therapy.^4^ As of today, the treatment with TKI is continued indefinitely in most patients. As a result, a growing number of more patients will be on a TKI for extended periods of time, which weighs importance on QoL.

Considering these prolonged treatment needs, we aimed to understand the impact this long‐standing therapy may have in the QoL of patients with CML. Focusing in QoL has multiple implications, not only for the well‐being of the patient, but also for the adherence to therapy and ultimately their response, as multiple treatment interruptions can decrease the probability of achieving the deepest molecular responses that we seek to consider treatment discontinuation.^22^ Some studies have suggested that patients with higher baseline symptom scores have a higher probability of treatment interruptions and hospitalizations, which eventually manifest as a delay in achieving a molecular response.^2^, ^7^, ^23^, ^24^ In our population, however, the majority of patients reached optimal treatment goals with minimal dose reductions, with the notable exception of the ponatinib cohort, although in this instance, dose reductions were preemptive secondary to FDA warning on the risk of increased cardiovascular events.^12^, ^24^ In fact, ultimately after an average of 18 months of therapy, patients were required to switch to another TKI. For this study, we used the MDASI questionnaire that has been developed and validated specifically for CML at our institution. Other tools have been used in CML in other studies. It would be important to confirm and/or complement our findings using other tools.

In all cohorts, fatigue was the most frequent symptom reported and interference with work the most affected activity. The interlinking of these two variables is not uncommon and has been reported in other series.^25^, ^26^ The resulting comparison between responder and non‐responder groups showed no significant difference, suggesting the disease has little impact on the symptoms. Symptom and interference scores improved over time.

Efficace et al^25^ have reported myalgia and fatigue were the symptoms most strongly affecting quality of life among patients treated with imatinib. We did not find such correlation in our series. In all individual cohorts, myalgia was of mild intensity and did not make it to the overall top 5 symptoms, showing a low trend for correlation with fatigue. This is not unexpected as imatinib (the only TKI included in the study by Efficace et al, and not included in our study) is known to cause more myalgias and muscle cramps than the three TKIs investigated in our series, that is, dasatinib, nilotinib, or ponatinib. This underscores how different symptoms that are characteristic of the different drugs may impact the total symptom score ultimately having similar general effects in quality of life. The two studies also used different assessment instrument, and we cannot rule out the possibility that this may have impacted the findings. Still, both studies show the important effect of fatigue on QoL, regardless of TKI.

We analyzed also the correlation between dose adjustments and symptom score. We identified different patterns by individual drug. The dasatinib cohort reported better mean scores among those who reduced doses later during the course of therapy. In contrast, for patients treated with nilotinib higher symptom scores were identified among patients with dose reductions at any time. For the ponatinib cohort, a lower score for skin rash correlated with reduced doses, but an opposite trend was seen for fatigue. The correlation between dose and symptom burden may be impacted by multiple factors. Although a high symptom burden might be a reason for dose reduction, the correlation may be only coincidental. This study was not designed to assess a cause‐effect correlation; thus, no conclusions can be derived from these observations, which should be further prospectively explored.

One important observation in our analysis over time is that symptoms for dasatinib and ponatinib cohorts including fatigue, skin rash, and WAW showed a peak around 9 months and later improved over time below baseline values by the end of the observation period. The improvement over time after this peak could be explained by a number of factors including the favorable therapeutic effect of TKI, the management of adverse events including dose adjustments, and possible habituation of the patient to the presence of chronic adverse events. In fact, several adverse events such as myelosuppression and liver function test abnormalities improved over time and even resolved spontaneously in some patients. Further assessments beyond 24 months would be warranted to explore how symptom scores may evolve in the longer term.

It is also important to note that baseline mean values are elevated for most symptoms. This probably reflects the effects of the disease itself and the anxiety generated by the recognition of a new devastating diagnosis that causes stress and apprehension to most patients. Over time, the effect, real or perceived, of the medication causing or contributing to these QoL measures becomes more relevant as the disease achieves optimal control and is not likely to contribute in a meaningful way to the presence of these symptoms. It is possible to speculate that some of the peaks that occur later may represent a transition from these early stages, to one where the stress caused by the initial diagnosis is relived at least in part as patients appreciate the good response to therapy, and some of the low‐grade but chronic adverse events accompanying therapy with TKI become more evident.

It is impossible to address the extent to which TKIs cause or contribute to symptoms such as fatigue, which are frequently multifactorial and common occurrences in daily life for any individual. Recent observations from discontinuation studies have suggested that QoL measures may not significantly improve upon TKI discontinuation.^27^, ^28^ This underscores the multifactorial etiology of some of the symptoms experienced by patients during TKI therapy. In any case, if the events are caused by TKI or multifactorial with TKI being only a partial contributor, understanding and managing these symptoms should become an important element of the management of patients with CML.

One other important factor is whether a change in therapy might be indicated to manage some of the symptoms associated with chronic TKI therapy. Several studies have shown that there is minimal cross‐intolerance between different TKIs.^29^ Unfortunately, the focus on these studies has been mostly on the more objective adverse events such as myelosuppression, liver function test abnormalities. Change in therapy for chronic, lower intensity symptoms has been investigated.^30^ Among patients treated with imatinib, nearly two thirds of symptoms improved after switching to nilotinib and this was accompanied by an improvement in QoL measures. Still, some patients experienced new adverse events and a few even had to discontinue nilotinib because of intolerable new events. Thus, although a change in therapy may help in many instances, it may come with new adverse consequences. This strategy has to be carefully reviewed with the patient with clear description of potential new adverse events to manage expectations that in some instances might be unrealistic.

In conclusion, patients treated with TKI experience chronic adverse events, mostly mild to moderate in severity, that affect their quality of life and interfere with daily activities. These scores peaked between 6 and 9 months from the start of therapy and improved gradually over time, but remain present at least up to 24 months after the start of therapy. Further studies should address the factors associated with these symptoms and interventions that may improve the patients’ QoL, including treatment discontinuation when safely feasible.

## CONFLICT OF INTEREST

HK received research grants from Novartis, Bristol‐Myers Squibb, and Ariad. EJ received consultancy fees for Ariad and Bristol‐Myers Squibb. JEC received research support from Ariad, Bristol‐Myers Squibb, and Novartis, and is a consultant for Ariad, Bristol‐Myers Squibb, and Novartis.
