# Supplementary material for: A prospective analysis of symptom burden for patients with chronic myeloid leukemia in chronic phase treated with frontline second‐ and third‐generation tyrosine kinase inhibitors
Source: Cancer Med. 2018 Oct 14;7(11):5457–69. doi: 10.1002/cam4.1808 (PMC6246941; doi:10.1002/cam4.1808)
Supplement: Supplementary file 1 [file CAM4-7-5457-s001.docx]

**SUPLEMENTAL MATERIAL**

**Supplemental Table 1**. Overall response by PCR IS transcript levels during 24 months of therapy.

|  | Months | | | | | |
| --- | --- | --- | --- | --- | --- | --- |
| PCR IS % Transcripts | **3**  **N=218**  **n(%)** | **6**  **N=213**  **n(%)** | **9**  **N=208**  **n(%)** | **12**  **N=204**  **n(%)** | **18**  **N=200**  **n(%)** | **24**  **N=193**  **n(%)** |
| < 0.00001 | 0 | 23 (10.7) | 28 (13.4) | 37 (18.1) | 47 (23.5) | 59 (30.5) |
| <0.0032 | 0 | 28 (13.1) | 29 (14) | 43 (21) | 49 (25) | 59 (30.5) |
| <0.01 | 17 (7.7) | 83 (39) | 88 (42.3) | 102 (50) | 120 (60) | 132 (68.3) |
| <0.1 | 81 (37.15) | 154 (72) | 157 (75) | 160 (78) | 169 (84) | 191 (98.9) |
| <10 | 184 (84) | 207 (97) | 199 (95) | 193 (96) | 186 (93) | 182 (94) |

**Supplemental Table 2.** Correlation between mean scores for the top 10 symptoms and mean percentage dose by 24 months from baseline for all patients and by treatment.

|  | Overall (obs=814) | | Dasatinib (obs=382) | | Nilotinib (obs=177) | | Ponatinib (obs=263) | |
| --- | --- | --- | --- | --- | --- | --- | --- | --- |
|  |  |  |  |  |  |  |  |  |
|  | ***r*** | ***P*** | ***r*** | ***P*** | ***r*** | ***P*** | ***r*** | ***P*** |
| Fatigue | -0.004 | 0.89 | 0.19 | 0.0001 | -0.10 | 0.15 | 0.01 | 0.85 |
| Drowsiness | -0.02 | 0.55 | 0.17 | 0.0007 | -0.18 | 0.01 | 0.02 | 0.73 |
| Sleep disturbance | -0.03 | 0.58 | 0.13 | 0.01 | -0.16 | 0.03 | 0.04 | 0.51 |
| Skin Rashes | -0.06 | 0.05 | 0.15 | 0.003 | -0.24 | 0.001 | 0.02 | 0.67 |
| Difficulty Remembering | 0.0003 | 0.99 | 0.17 | 0.0005 | -0.15 | 0.03 | 0.01 | 0.86 |
| Muscle Cramps | -0.12 | 0.002 | 0.08 | 0.12 | -0.19 | 0.009 | -0.12 | 0.04 |
| Distress | -0.006 | 0.55 | 0.12 | 0.01 | -0.08 | 0.3 | 0.08 | 0.17 |
| Pain | -0.03 | 0.28 | 0.09 | 0.07 | -0.09 | 0.21 | -0.01 | 0.75 |
| Malaise | -0.003 | 0.92 | 0.12 | 0.01 | 0.01 | 0.81 | 0.01 | 0.86 |
| Dry mouth | -0.11 | 0.001 | 0.05 | 0.31 | -0.21 | 0.005 | -0.02 | 0.64 |

OBS=Observations. Cohorts analyzed separately showed medium to large effect sizes (r>0.3) (see table 5).
